# USP46 Inhibits Cell Proliferation in Lung Cancer through PHLPP1/AKT Pathway

**DOI:** 10.1155/2020/2509529

**Published:** 2020-09-23

**Authors:** Wei Wang, Meng Chen, Hailing Xu, Dongqing Lv, Suna Zhou, Haihua Yang

**Affiliations:** ^1^Laboratory of Cellular and Molecular Radiation Oncology, Radiation Oncology Institute of Enze Medical Health Academy, Affiliated Taizhou Hospital of Wenzhou Medical University, Taizhou, 317000 Zhejiang Province, China; ^2^Department of Radiation Oncology, Affiliated Taizhou Hospital of Wenzhou Medical University, Taizhou, 317000 Zhejiang Province, China; ^3^School of Medicine, Shaoxing University, Shaoxing City, 312000 Zhejiang Province, China; ^4^Department of Pulmonary Medicine, Enze Hospital, Affiliated Taizhou Hospital of Wenzhou Medical University, Taizhou, 317000 Zhejiang Province, China

## Abstract

Previous studies have shown that ubiquitin-specific protease 46 (USP46) is a tumor suppressor in colon cancer and renal cell carcinoma. However, its specific role in other cancers is still poorly understood. This study is aimed at investigating the role of USP46 in lung cancer tumorigenesis and identifying its underlying mechanisms. Quantitative real-time polymerase chain reaction (qRT-PCR) and western blotting (WB) were used to measure the expression levels of USP46 and PHLPP1 in lung cancer tissue and adjacent normal tissue from patients with lung cancer. We examined the ability of USP46 to regulate cell proliferation in lung cancer cells via cell proliferation assay, radiation assay, genetic overexpression and knockdown, and chemical inhibition of relevant genes. We investigated the underlying mechanisms in multiple lung cancer cell line models by coimmunoprecipitation and ubiquitination assays. In this study, we identified a strong downregulation of the expressions of USP46 and PHLPP1 in lung cancer tissues relative to normal adjacent tissues. USP46 was further shown to inhibit lung cancer cell proliferation under conditions of normal growth and during radiation-induced DNA damage by antagonizing the ubiquitination of PHLPP1 resulting in the inhibition of AKT signaling. Exposure to radiation and AKT inhibition significantly reversed the effect of USP46 siRNA on lung cancer cell proliferation. USP46 is downregulated in lung cancer and suppresses the proliferation of lung cancer cells by inhibiting the PHLPP1/AKT pathway. AKT inhibition slows the proliferation of lung cancer cells that have been downregulated by USP46 and exposed to radiation. This suggests a potential therapeutic avenue for USP46-downregulated lung cancer through a combination of radiation and AKT inhibitor treatment.

## 1. Introduction

Lung cancer is the most deadly form of cancer for the sexes and has one of the worst survival rates [1]. Every year, about 1.6 million people are estimated to die from lung cancer [2]. Recent advancements in cancer therapies have improved the survival rate of patients with lung cancer; however, there are still unmet needs in terms of therapies. A key step toward developing effective therapies against lung cancer is elucidating the molecular mechanisms underlying tumorigenesis.

Protein posttranslational modifications play important roles in regulating numerous biological processes, such as the cell cycle, DNA damage repair, and apoptosis [3]. Ubiquitination is one of the most ubiquitous posttranslational modifications and is dynamically regulated by ubiquitin ligases and deubiquitinase [4]. Ubiquitin-specific protease 46 (USP46) is a member of the cysteine protease family which functions as a deubiquitinase and has been associated with neurological disorders [5] and behavioral abnormalities [6]. The vital role of USP46 in regulating neuronal signaling may be the most probable cause of these phenotypes. For example, USP46 has been shown to regulate glutamate receptor (GLR) function by regulating the abundance of GLR-1 [7]. Likewise, USP46 has been shown to deubiquitinate alpha-amino-3-hydroxy-5-methyl-4-isoxazolepropionic acid receptors (AMPARs) leading to the stabilization of the receptor [8]. Recent studies have identified other substrates of USP46 that have important functions in cancer progression. However, the role of USP46 in lung cancer remains unclear.

USP46 had been shown to deubiquitinate the PH domain leucine-rich repeat protein phosphatase 1 (PHLPP1) in colorectal cancer [9]. The PHLPP1 ubiquitination primes its degradation, and deubiquitination thus protects PHLPP1 from degradation and enhances its activity in the cell [9]. One of the key substrates of PHLPP1 is Protein Kinase B (PKB, also called AKT). PHLPP1 removes activating phosphorylations in AKT proteins thus antagonizing the activity of AKT [10].

AKT family proteins are serine/threonine kinases that play critical roles in cell proliferation, DNA damage repair, and cell survival during conditions of stress [11]. In different types of cancers, AKT family genes are frequently mutated, and inhibitors against the kinases are used in the treatment of several cancers [12]. Previously, in colorectal cancer, USP46 had been shown to antagonize the activity of AKT by deubiquitinating PHLPP1 [9]. Consistent with the antiproliferation activity of USP46, the protein was found to be downregulated in colorectal cancer [9], suggesting that USP46 is an important tumor suppressor gene. USP46 has equally shown a tumor suppressor activity in renal cell carcinoma with a similar mechanism to that described in colorectal cancer [13]. However, the detailed relationship between USP46 and the PHLPP1/AKT pathway is still less investigated in lung cancer.

Herein, we investigated the potential role of USP46 in lung cancer tumorigenesis and found that the expressions of USP46 and PHLPP1 were downregulated in lung cancer. Mechanistically, we found that USP46 inhibits cell proliferation in lung cancer cells by inhibiting the AKT pathway, in part by preventing DNA damage repair.

## 2. Materials and Methods

### 2.1. Human Tissue Samples

Thirty pairs of lung cancer tissues and adjacent-matched normal tissues were used to investigate mRNA and protein levels of different genes. All patients provided informed and written consent. The independent ethics committee of the Affiliated Taizhou Hospital of Wenzhou Medical University approved this study, and it was in accordance with the Declaration of Helsinki.

### 2.2. Cell Culture

Cell lines (A549, H358, H446, H1299, and 16HBE) were obtained from the cell bank of the Shanghai Biology Institute (Shanghai, China). Cells were cultured in Dulbecco's modified Eagle's medium (Trueline, Kaukauna, USA) supplemented with 10% fetal bovine serum (Thermo Fisher Scientific), 2 mM L-glutamine, and 1% penicillin/streptomycin (Solarbio, Beijing, China) and maintained under 5% CO_2_ atmosphere at 37°C. The AKT inhibitor LY294002 (25 *μ*mol/L; S1105, Selleck, USA) was dissolved in DMSO (D2650, Sigma, USA) and added to the cell culture.

### 2.3. Quantitative Real-Time Polymerase Chain Reaction (qRT-PCR)

RNAs were isolated using a TRIzol reagent (Invitrogen, Waltham, USA). cDNA was synthesized by using a cDNA synthesis kit (Thermo Fisher Scientific, Waltham, USA) following the instructions of the manufacturer. The thermal cycle was set as follows: 95°C for 10 min followed by 40 cycles of 95°C for 15 s and 60°C for 45 s. All data represent an average of three replicates. The primers used were as follows: USP46 (NM_001134223.2): forward primer: 5′-AGAAGCCCAGAAAAGGATGAGG-3′, reverse primer: 5′-CAAAAGCCAGAAGCCGTGAC-3′; PHLPP1 (NM_194449.4): forward primer: 5′-AATGTGCCTGAGTGGGTATGTG-3′, reverse primer: 5′-TCATCAGAA GGTTAGGTGGGAG-3′; and beta-actin (HQ154074.1): forward primer: 5′-CGTGGACATCCGCAAAGAC-3′, reverse primer: 5′-TGCTGGGAGCCAGAGCAG-3′.

### 2.4. Western Blotting (WB)

A radioimmunoprecipitation assay (RIPA) lysis buffer (JRDUN, Shanghai, China) containing an EDTA-free protease inhibitor cocktail (Roche, Heidelberg, Germany) was used to extract whole protein lysates, followed by protein concentration measurement by an enhanced BCA protein assay kit (Thermo Fisher Scientific). Twenty-five micrograms of proteins from each sample was run in 10% SDS-PAGE gel and transferred to a nitrocellulose membrane (Millipore, Billerica, USA) overnight. After blocking with 5% nonfat dry milk for 1 h at room temperature, the membranes were probed at 4°C overnight with primary antibodies followed by secondary anti-mouse IgG antibody (1 : 1,000; Beyotime, Shanghai, China) for 1 h at 37°C. An enhanced chemiluminescence system (Tanon, Shanghai, China) was used to visualize protein expressions. Detailed information of primary antibodies is as follows: anti-USP46 (Ab88795, Abcam, UK), anti-PHLPP1 (Ab135957, Abcam, UK), anti-p-AKT (#9271, CST, USA), anti-AKT (#9272, CST, USA), and anti-*β*-actin (#4970, CST, USA).

### 2.5. Knockdown and Overexpression of USP46

Lentiviral plasmids (pLKO.1) containing three siRNAs directed to different regions of human USP46 (NM_001134223.2) and a negative control siRNA (siNC) were synthesized (Major, Shanghai, China). The USP46 overexpression plasmid was constructed by inserting the full-length human USP46 cDNA sequence into a lentiviral plasmid (pLVX-puro), while the vector plasmid was used as a negative control (oeNC). Experiments were performed 48 h after the transit transfection of the plasmids into lung cancer cells using Lipofectamine 2000 (Thermo Fisher Scientific) following the instructions of the manufacturer. Detailed nucleotide sequences are as follows: siUSP46-1: (8–26; TCCGAAACATCGCCTCCAT), siUSP46-2: (17–35; TCGCCTCCATCTGTAATAT), and siUSP46-3 (26–44, TCTGTAATATGGGCACCAA).

### 2.6. Cell Proliferation Assay

Cell proliferation was analyzed by Cell Counting Kit-8 (CCK-8) (SAB, College Park, USA) following the instructions of the manufacturer. Briefly, cells were seeded in 96-well plates for 0, 24, 48, and 72 h. CCK-8 solution (1 : 10) was added to each well and incubated for 1 h. The absorbance was measured by the microplate reader (Pulangxin, Beijing, China) at 450 nm.

### 2.7. Immunofluorescence (IF)

Cells were fixed with 4% paraformaldehyde for 30 min at room temperature. After that, samples were washed with PBS three times, for 3 min each time, at 25°C and blocked with 1% BSA (Solarbio, Beijing, China) for 1 h at room temperature. Subsequently, cells were incubated with rabbit anti-*γ*-H2AX antibody (ab2893, Abcam, UK) in PBS overnight at 4°C followed by goat anti-rabbit IgG (H+L) (A0423, Beyotime, Haimen, China) for 1 h at room temperature. Images were obtained by an ECLIPSE Ni microscope and a digital image analyzer (Nikon, Tokyo, Japan).

### 2.8. Coimmunoprecipitation (co-IP)

For IP, whole-cell extracts were prepared after transfection or stimulation with appropriate ligands; the lysates were incubated overnight with the appropriate antibodies conjugated to Protein A/G beads (Santa Cruz Biotechnology, USA). Beads were washed five times and separated by western blotting as described above.

### 2.9. Ubiquitination Assay

After 48 h of transfection with oeNC or oeUSP46, the cells were lysed with 1% SDS-containing RIPA buffer and sonicated. Subsequently, samples were incubated with IgG (sc-2027, Santa Cruz Biotechnology, USA) or PHLPP1 antibody (PA5-34434, Invitrogen, USA) overnight at 4°C, followed by incubation with Protein A/G PLUS-Agarose (sc-2003, Santa Cruz Biotechnology, USA) for 1 hour. The beads were centrifuged at 3,000 rpm for 5 min at 4°C and subsequently washed with wash buffer four times. The purified proteins were separated by 4%–20% gradient SDS-PAGE protein gel and immunoblotted with antiubiquitin antibody (ab7780, Abcam, UK).

### 2.10. Data Analysis

Data are presented as mean ± standard deviation (SD) based on three independent experiments. Statistical significance was determined by one-way ANOVA for multiple comparisons using GraphPad Prism software version 7.0 (USA). A *p* value < 0.05 was considered statistically significant.

## 3. Results

### 3.1. Both USP46 and PHLPP1 Were Downregulated in Human Lung Cancer Tissues

**Figure 1:**
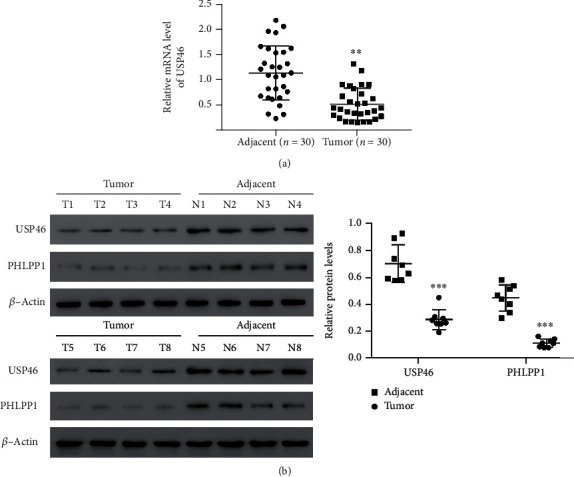
USP46 and PHLPP1 were downregulated in human lung cancer tissues. (a) mRNA level of USP46 in human lung cancer and adjacent-matched tissues was measured by qRT-PCR. USP46 mRNA levels were normalized to the beta-actin mRNA levels (*n* = 30). ∗∗ means *p* < 0.01. (b) Expressions of USP46 and PHLPP1 in 8 pairs of human lung cancer and adjacent-matched tissues were measured by western blotting (left), and protein levels were quantified according to the gray value after normalization to *β*-actin (*n* = 8). ∗∗∗ means *p* < 0.001.

Using qRT-PCR, the expression of USP46 was measured in lung cancer tissues and adjacent normal tissues in 30 patients. We found that cancer tissues had significantly lower levels of USP46 messenger RNA (mRNA) (Figure 1(a)). The protein level of USP46 was further evaluated in the lung cancer tissues of 8 patients, and as expected, the lower level of USP46 mRNA in the cancer tissue compared to the adjacent normal tissue correlated with the protein level (Figure 1(b)). We also compared the expression of USP46 in several human lung cancer cell lines to normal human bronchial epithelial (HBE) cells and found that USP46 expression was greatly decreased both in mRNA levels (Figure S1A) and protein levels (Figure S1B) in the lung cancer cell lines compared to HBE cells.

In colorectal cancer, USP46 has been shown to increase the stability of PHPLL1 [9]; hence, we investigated the protein levels of USP46 and PHPLL1 in lung cancer tissues. In effect, both USP46 and PHPLL1 were downregulated in lung cancer tissues compared to the adjacent cancer tissues (Figure 1(b)). Therefore, present results suggested that USP46 might be positively correlated with PHLPP1 in lung cancer tissues.

### 3.2. USP46 Inhibited the Proliferation of Human Lung Cancer Cells

**Figure 2:**
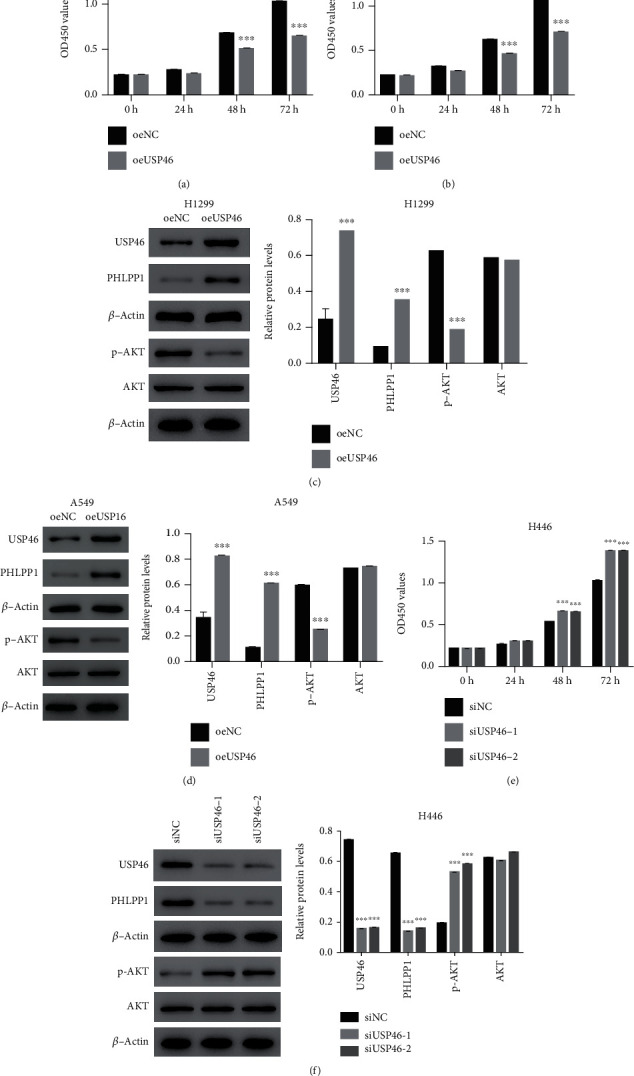
USP46 inhibited proliferation of human lung cancer cells. (a, b) Cell proliferation was measured by CCK-8 in H1299 cells (a) and A549 cells (b), and the rate of proliferation was compared between cells overexpressing USP46 (oeUSP46) and control cells (oeNC) (*n* = 3). ^∗∗∗^ means *p* < 0.001. (c, d) Protein levels or phosphorylation levels of indicated proteins were measured by WB in H1299 cells (c) and in A549 cells (d) overexpressing USP46 (oeUSP46) cells and control (oeNC) cells, and their quantifications are shown (*n* = 3). ∗∗∗ means *p* < 0.001. (e) Cell proliferation in H446 cells transfected with control siRNA (siNC) or USP46 siRNA (siUSP46-1 and siUSP46-2) was measured by CCK-8 (*n* = 3). ∗∗∗ means *p* < 0.001. (f) Levels of indicated proteins and phosphorylations were measured by WB in cells shown in (e), and their quantifications are shown (*n* = 3). ∗∗∗ means *p* < 0.001.

Subsequently, we evaluated the role of USP46 in the cell proliferation of several lung cancer cell lines. We overexpressed USP46 in H1299 and measured their proliferation. The cells overexpressing USP46 (oeUSP46) showed significantly lower proliferation 48 h after USP46 overexpression compared to the control cells (oeNC) (Figure 2(a)). The inhibition of proliferation was even more prominent after 72 h posttransfection (Figure 2(a)). To verify that the effect of USP46 in proliferation is a general phenomenon in lung cancer cells, we repeated the experiment in another lung cancer cell line, A549. As expected, oeUSP46 inhibited proliferation in the A549 cell line as well (Figure 2(b)).

Subsequently, we sought to identify potential mechanisms through which USP46 regulates cell proliferation in lung cancer cells. In colorectal cancer, USP46 has been shown to promote the stabilization of PHLPP1 and subsequent inhibition of the AKT pathway [9]. Hence, we examined the protein level of PHLPP1 and the activation status of AKT in H1299 oeUSP46 cells (Figures S1C and S1D and Figure 2(c)). Indeed, oeUSP46 led to a significant increase in the PHLPP1 protein level and a significant decrease in the activating AKT phosphorylation without altering the protein level of AKT (Figure 2(c)), demonstrating the role of USP46 in increasing the protein level of USP46 and inhibiting the AKT signaling pathway. Similar results were equally observed in A549 cells (Figure S1C and S1D and Figure 2(d)). Conversely, the knockdown of USP46 led to significant increase in the proliferation of H446 cell lines (Figures S1E and S1F and Figure 2(e)), as well as a decrease in PHLPP1 protein levels and an increase in the activation of AKT phosphorylation (Figure 2(f)). Collectively, these results established the role of USP46 in inhibiting cell proliferation in lung cancer cells by promoting the stability of USP46 and subsequently inhibiting the AKT pathway.

### 3.3. USP46 Promoted PHLPP1 Protein Stability via Deubiquitination in Human Lung Cancer Cells

**Figure 3:**
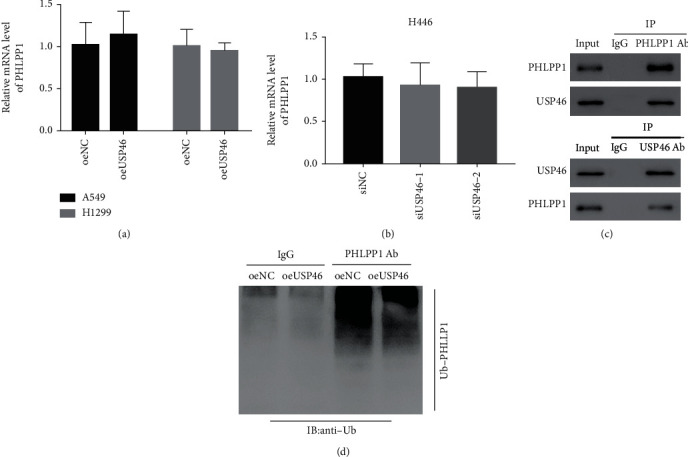
USP46 promoted PHLPP1 protein stability via deubiquitination in human lung cancer cells. (a, b) mRNA levels of PHLPP1 in A549 and H1299 cells upon USP46 overexpression (a) and USP46 knockdown (b) were measured by RT-PCR. The data were normalized to beta-actin. Mean ± standard deviation (SD) (*n* = 3). (c) Interaction between USP46 and PHLPP1 was tested by co-IP followed by WB using indicated antibodies. (d) USP46-dependent ubiquitination in PHLPP1 was measured by comparing ubiquitination in PHLPP1 in USP46 overexpressing cells (oeUSP46) and control cells (oeNC).

We investigated the mechanism through which USP46 causes an increase in PHLPP1 protein levels. First, we examined if USP46 regulates the expression of PHLPP1 in the transcriptional level. We found that neither overexpression nor knockdown of USP46 has any effect in the mRNA level of PHLPP1 (Figures 3(a) and 3(b)). These results suggested a potential regulation in the protein level. Hence, we checked physical interaction between USP46 and PHLPP1 by coimmunoprecipitation (co-IP). We found that PHLPP1 precipitated with USP46 and vice versa (Figures 3(c)) demonstrating that USP46 and PHLPP1 physically interact with each other.

USP46 is a deubiquitinase, so we hypothesized that USP46 deubiquitinates PHLPP1 in lung cancer cells. To test this hypothesis, we overexpressed USP46 and measured the level of ubiquitination in PHLPP1 in H1299 and A549 cells. Compared to the control cells, the overexpression of USP46 led to a decrease in ubiquitination in PHLPP1 (Figure 3(d)), indicating that USP46 deubiquitinates PHLPP1 in lung cancer cell lines.

### 3.4. USP46 Inhibited Cell Proliferation during DNA Damage in Human Lung Cancer Cells

AKT family kinases play an important role in DNA damage repair [14]. More importantly, recent studies have suggested that AKT activity is a good predictive marker of radiation response in cancer treatment and that AKT promotes postradiation cell survival by accelerating DNA damage repair (IR) [15–17].

**Figure 4:**
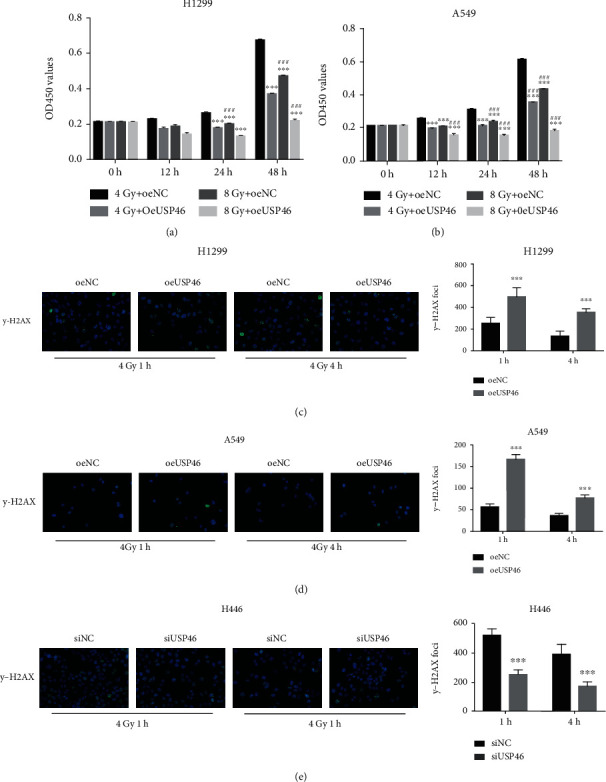
USP46 inhibited cell proliferation during DNA damage in lung cancer cells. (a, b) Proliferation of H1299 (a) and A549 (b) cells overexpressing USP46 (oeUSP46) and control vectors (oeNC) exposed to radiation was measured by CCK-8. Mean ± standard deviation (SD) (*n* = 3). ∗∗∗ means *p* < 0.001 (vs. 4 Gy+oeNC); ### means *p* < 0.001 (vs. 4 Gy+oeUSP46). (c, d) *γ*-H2AX foci formation in H1299 (c) and A549 (d) oeNC and oeUSP46 cells after exposure to radiation was assessed by IF at the indicated time points after 4 Gy radiation exposure. (e) *γ*-H2AX foci formation in USP46 knockdown H446 cells after exposure to 4 Gy radiation was assessed by IF.

Hence, we investigated if USP46 is involved in the inhibition of cell proliferation following DNA damage. We treated cells with varying degrees of ionizing radiation (IR) to induce DNA damage and measured cell proliferation at different time points post-IR. oeUSP46 cells showed significantly less cell proliferation compared to the control cells in both H1299 and A549 cells (Figures 4(a) and 4(b)). The degree of reduction in cell proliferation in oeUSP46 cells correlated with the degree of IR (Figures 4(a) and 4(b)).

DNA damage in the cells induces phosphorylation at the Ser139 residue of histone H2AX forming gamma H2AX (*γ*-H2AX) foci; hence, *γ*-H2AX can be used as a marker of DNA damage in the cells [18]. Since PHLPP1 inhibits the activation of AKT, we investigated the effect of USP46 expression in the formation of DNA damage-dependent *γ*-H2AX foci upon exposure of cells to IR. The overexpression of USP46 led to an increase in *γ*-H2AX foci formation in both H1299 and A549 cells 1 h post-IR (Figures 4(c) and 4(d)), and the foci formation was not restored even 4 h post-IR (Figures 4(c) and 4(d)). Conversely, the knockdown of USP46 showed a decrease in *γ*-H2AX foci formation post-IR (Figure 4(e)). Collectively, these results indicate the role of USP46 in inhibiting lung cancer cell proliferation during DNA damage.

### 3.5. AKT Inhibition Improved the Proliferation Rate of USP46 Knockdown Cells Exposed to Radiation

**Figure 5:**
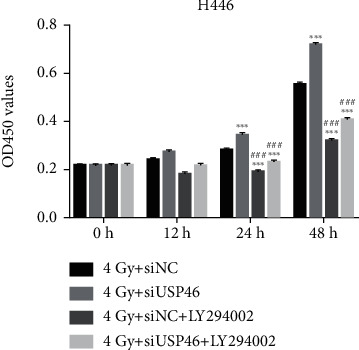
AKT inhibition improved the proliferation rate of USP46 knockdown cells exposed to radiation. Control cells (siNC) or USP46 knockdown cells (siUSP46) exposed to 4 Gy radiation were treated with LY294002, and cell proliferation was measured by CCK-8. Mean ± standard deviation (SD) (*n* = 3). ∗∗∗ means *p* < 0.001 (vs. 4 Gy+siNC); ### means *p* < 0.001 (vs. 4 Gy+siUSP46).

Subsequently, we tested the effect of an AKT inhibitor, LY294002, in lung cancer cell proliferation during DNA damage and checked the role of USP46 in this context. As expected, we found that knockdown of USP46 increased the proliferation of H446 cells following exposure to IR (Figure 5). However, the proliferation of the cancer cells was significantly reduced when AKT kinases were inhibited by LY294002 (Figure 5). The inhibition in proliferation was partially rescued in the cells treated with LY294002 and exposed to IR in the USP46 knocked down cells (Figure 5). Collectively, these results suggest an important role of USP46 in antagonizing the activity of AKT in cell proliferation following DNA damage.

## 4. Discussion

In this study, we showed that USP46 expression is downregulated in patients with lung cancer, suggesting a tumor suppressor role of USP46 in lung cancer, which is consistent with the reports from other studies in colorectal cancer [9] and renal cell carcinoma [13]. Functionally, we demonstrated that USP46 inhibits cell proliferation in lung cancer cells under normal growth conditions and in cells exposed to IR. Conversely, we showed that the downregulation of USP46 increases the proliferation of lung cancer cells, and exposure to radiation in combination with an AKT inhibitor suppresses the proliferation of the lung cancer cells. These results have huge clinical significance as they indicate a potential therapeutic avenue in USP46-downregulated lung cancers through a combination therapy involving radiation and AKT inhibitors.

Mechanistically, we showed that the antiproliferation activity of USP46 in lung cancer is mediated through the inhibition of AKT activity via deubiquitination of PHLPP1. These findings form a solid foundation for *in vivo* experiments where the precise role of USP46 in lung cancer progression can be evaluated in more physiological conditions in mouse models. It will be interesting to investigate if USP46 is primarily involved in the primary tumor growth or if it is important for metastasis or both. These questions will be the subject of future studies.

Lung cancer is the most deadly cancer in both men and women in the United States with one of the lowest survival rates [19]. Although several treatment options are available for patients with lung cancer, there is still a huge unmet clinical need. Our study has identified USP46 as an important negative regulator of cell proliferation in lung cancer cells. We also established an adverse clinical correlation of USP46 expression in lung cancer patients. These findings suggest that targeting pathways or proteins that increase the expression of USP46 could be a beneficial therapeutic avenue for lung cancer treatment. Our study along with previous studies strongly suggests the tumor suppressor role of USP46 in multiple cancers. It will be interesting to investigate if the knockout of USP46 is sufficient to drive tumorigenesis by itself or in combination with other known mutations. Furthermore, it remains to be investigated if there are recurrent mutations in USP46 genes in patients with cancer and if, subsequently, they correlate with other known driver mutations. Based on our study, it is very likely that there will be some correlations with mutations in PI3K/AKT genes across multiple cancers. A thorough analysis of the genomic databases could easily provide more concrete answers to these important questions.

We also showed that USP46 antagonizes the activity of the AKT pathway that affects the proliferation of lung cancer cells. Multiple classes of potent AKT inhibitors have been developed, and some of them are already used in the clinical setting for different diseases and some are currently under clinical trials for the treatment of different cancers [20]. Drugs targeting AKT might benefit patients with lung cancer with lower expressions of USP46. It will be interesting to know if the patients who respond to the AKT inhibitors correlate with the expression levels of USP46. Future work will shed more light into the functional ties between USP46 and AKT during cancer therapies.

## 5. Conclusion

This study establishes an important role of USP46 in antagonizing the activity of AKT in lung cancer cells that ultimately inhibit cell proliferation. Mechanistically, we showed that USP46 stabilizes PHLPP1 by deubiquitinating the protein, and thus negatively affecting AKT activity. Clinically, we showed that USP46 and PHLPP1 are downregulated in patients with lung cancer.

## Data Availability

All data generated or analyzed during this study are included in this published article.

## Abbreviations

qRT-PCR: Quantitative real-time polymerase chain reaction
WB: Western blotting
USP46: Ubiquitin-specific protease 46
AMPARs: Alpha-amino-3-hydroxy-5-methyl-4-isoxazolepropionic acid receptors
PHLPP1: PH domain leucine-rich repeat protein phosphatase 1.

## Supplementary Materials

Supplementary materials Figure S1: expressiS1on of USP46 in human lung cancer cells was measured.(A, B) Relative mRNA and protein levels of USP46 in normal human bronchial epithelium (HBE) cells and indicated human lung cancer cells were measured by qRT-PCR (A) and WB (B), respectively, and the protein levels were quantified. Mean ± standard deviation (SD) (*n* = 3), ^∗∗∗^ means *p* < 0.001. (C, D) Relative mRNA and protein levels of USP46 in indicated human lung cancer cells overexpressing USP46 were measured by RT-PCR (C) and WB (D), respectively, and the protein levels were quantified. USP46 mRNA level was normalized to beta-actin mRNA. Mean ± SD (*n* = 3), ^∗∗∗^ means *p* < 0.001. (E, F) Relative mRNA and protein levels of USP46 in H446 cells upon USP46 knockdown were measured by qRT-PCR (E) and WB (F), respectively, and the protein levels were quantified. Mean ± SD (*n* = 3), ^∗∗∗^ means *p* < 0.001.
