# Supplementary material for: USP46 Inhibits Cell Proliferation in Lung Cancer through PHLPP1/AKT Pathway
Source: Biomed Res Int. 2020 Sep 23;2020:2509529. doi: 10.1155/2020/2509529 (PMC7532402; doi:10.1155/2020/2509529)
Supplement: Supplementary materials — Figure S1: expressiS1on of USP46 in human lung cancer cells was measured.(A, B) Relative mRNA and protein levels of USP46 in normal human bronchial epithelium (HBE) cells and indicated human lung cancer cells were measured by qRT-PCR (A) and WB (B), respectively, and the protein levels were quantified. Mean ± standard deviation (SD) (n = 3), ∗∗∗ means p < 0.001. (C, D) Relative mRNA and protein levels of USP46 in indicated human lung cancer cells overexpressing USP46 were measured by RT-PCR (C) and WB (D), respectively, and the protein levels were quantified. USP46 mRNA level was normalized to beta-actin mRNA. Mean ± SD (n = 3), ∗∗∗ means p < 0.001. (E, F) Relative mRNA and protein levels of USP46 in H446 cells upon USP46 knockdown were measured by qRT-PCR (E) and WB (F), respectively, and the protein levels were quantified. Mean ± SD (n = 3), ∗∗∗ means p < 0.001. [file 2509529.f1.pdf]

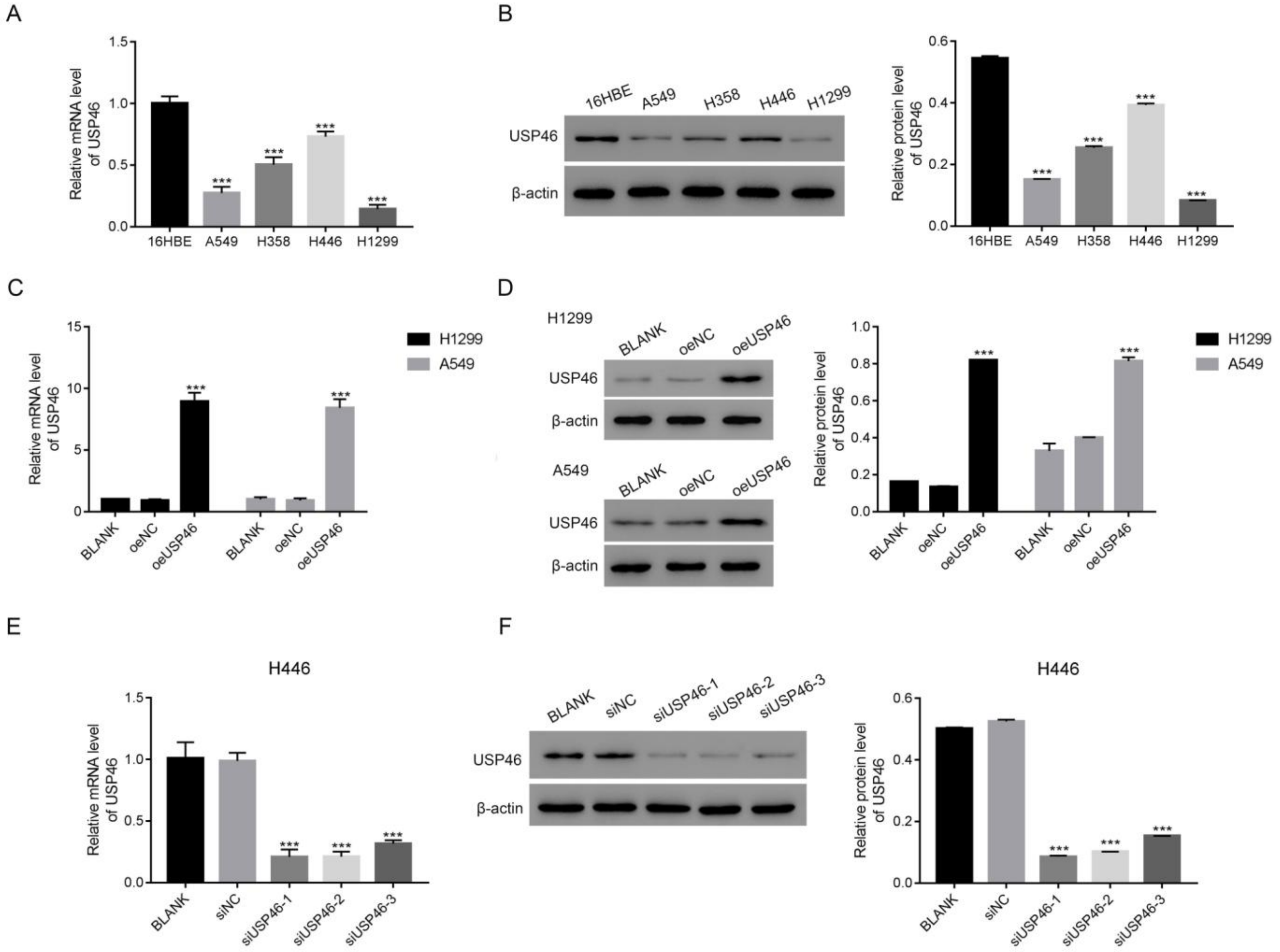

Figure S1: Expression of USP46 in human lung cancer cells was measured. A & B**.** Relative mRNA and protein levels of USP46 in normal human bronchial epithelium (HBE) cells and indicated human lung cancer cells were measured by qRT-PCR (A) and WB (B) respectively, and the protein levels were quantified. Mean ± SD, (n=3), *** means p < 0.001. C & D. Relative mRNA and protein levels of USP46 in indicated human lung cancer cells overexpressing USP46 were measured by RT-PCR (C) and WB (D) respectively, and the protein levels were quantified. USP46 mRNA level was normalized to beta-actin mRNA. Mean ± SD, (n=3), *** means p < 0.001. E & F. Relative mRNA and protein levels of USP46 in H446 cells upon USP46 knock down were measured by qRT-PCR (E) and WB (F) respectively, and the protein levels were quantified. Mean ± SD, (n=3), *** means p < 0.001.
